# WELL-BEING DURING THE COVID-19 PANDEMIC: A FUNCTION OF PERSONALITY, AGE, AND URBANICITY

**DOI:** 10.1093/geroni/igac059.892

**Published:** 2022-12-20

**Authors:** Lindsay Ryan

**Affiliations:** The University of Michigan, Ann Arbor, Michigan, United States

## Abstract

The COVID-19 pandemic has differentially impacted population sub-groups over the last two years. For example, engagement in social isolation may have been particularly difficult for extroverted adults. The virus spread widely in densely populated regions and is more risky to health with increasing age. This paper explores the ways in which personality, age, and urbanicity are associated with subjective well-being during the pandemic. Longitudinal data from the Health and Retirement Study (N = 4316; M age = 69.0, Range 31 - 99) investigates Big Five personality characteristics from 2016 and interactions with age on life satisfaction and loneliness during the pandemic. Models are then stratified by Beale Rural-Urban Continuum codes denoting urban, suburban, and ex-urban residence. Results indicate the benefit of high conscientiousness on life satisfaction is weaker among older adults (p<.05) and associations of extroversion and age on loneliness are driven by individuals living in urban areas (p<.05).

